# Association between the Standardized Mortality Ratio and Healthy Life Expectancy in Japan

**DOI:** 10.31662/jmaj.2022-0140

**Published:** 2022-12-19

**Authors:** Rikuya Hosokawa, Toshiyuki Ojima, Tomoya Myojin, Jun Aida, Katsunori Kondo, Naoki Kondo

**Affiliations:** 1 Department of Human Health Sciences, Graduate School of Medicine, Kyoto University, Kyoto, Japan; 2 Department of Community Health and Preventive Medicine, Hamamatsu University School of Medicine, Shizuoka, Japan; 3 Department of Public Health, Health Management and Policy, Nara Medical University, Nara, Japan; 4 Department of Oral Health Promotion, Graduate School of Medical and Dental Sciences, Tokyo Medical and Dental University, Tokyo, Japan; 5 Center for Preventive Medical Sciences, Chiba University, Chiba, Japan; 6 Center for Well-being and Society, Nihon Fukushi University, Aichi, Japan; 7 Center for Gerontology and Social Science, Research Institute, National Center for Geriatrics and Gerontology, Aichi, Japan; 8 School of Public Health and Graduate School of Medicine, Kyoto University, Kyoto, Japan

**Keywords:** life expectancy, mortality, healthcare, Japan, standardized mortality ratio

## Abstract

**Introduction:**

Healthy life expectancy (HLE) remains the principal target of various health plans. We aimed to identify the areas of priority and determinants of mortality to extend HLE across local governments in Japan.

**Methods:**

HLE according to secondary medical areas was calculated using the Sullivan method. People requiring long-term care of level 2 or higher were considered unhealthy. Standardized mortality ratios (SMRs) for major causes of death were calculated using vital statistics data. The association between HLE and SMR was analyzed using simple and multiple regression analyses.

**Results:**

The average (standard deviation) HLE values were 79.24 (0.85) and 83.76 (0.62) years for men and women, respectively. A comparison of HLE revealed regional health gaps of 4.46 (76.90-81.36) and 3.46 (81.99-85.45) years for men and women, respectively. The coefficients of determination for the SMR of malignant neoplasms with HLE were the highest and were 0.402 and 0.219 among men and women, respectively, followed by those of cerebrovascular diseases, suicide, and heart diseases among men and those of heart disease, pneumonia, and liver disease among women. When all major preventable causes of death were analyzed simultaneously in a regression model, the coefficients of determination were 0.738 and 0.425 among men and women, respectively.

**Conclusions:**

Our findings suggest that local governments should prioritize preventing cancer deaths via cancer screening and smoking cessation measures in health plans, with a special focus on men.

## Introduction

Life expectancy has improved worldwide over the last few decades ^(1)^. At the same time, an increasing number of people are experiencing functional decline as they age and spend more time living with comorbidities. Healthy life expectancy (HLE) is an important indicator for monitoring changes and assessing population health status. The measurement of HLE considers the number of years that an individual can spend in good health ^(1)^. LE is increasing not only in Japan but also in other countries; however, compared to the increase in years of life expectancy, the increase is small ^(2), (3), (4), (5), (6), (7)^. Therefore, the extension of HLE or the period of adult life without health limitations is a key consideration of Japan’s national health promotion program, Health Japan 21 (the second stage) ^(8), (9)^. Furthermore, HLE extension is considered the most important goal of health promotion strategies, such as the Healthy People 2030 in the United States and those conducted by the World Health Organization ^(10), (11), (12)^.

HLE is assessed using mortality rates and data from “healthy” and “unhealthy” citizens available at national public registries. Japan has been consistently ranked first for increased societal aging among the Organization for Economic Co-operation and Development (OECD) member countries ^(13), (14)^. Japan has the longest average life expectancy and HLE for both sexes; the longevity rate for women is particularly high. According to the World Health Organization, in 2019, Japan had the highest estimated life expectancy at birth at 81.5 and 86.9 years for men and women, respectively ^(13), (14)^. In addition, the HLE values in 2019 were 72.6 and 75.5 years for men and women, respectively, ranking Japan above all other countries worldwide ^(13), (14)^. However, the difference between life expectancy and HLE indicates the average number of years spent in poor health. This implies that Japanese people are functionally limited in their daily lives during these years. Although Japanese people have a long average life expectancy, it does not necessarily equate to having a high quality of life.

Several cases of functional limitations in daily life are caused by diseases and incidences that lead to death ^(15)^. Cancer or malignant neoplasm is the leading cause of death in Japan, accounting for 27% of all deaths in 2018, followed by heart disease at 15% ^(16)^. Globally, compared with those in other countries, the low mortality rates associated with cancer and ischemic heart disease remain conspicuous. The longevity of the Japanese population in recent years is a result of the low mortality rates associated with these diseases, accounting for nearly half of the total number of deaths. However, the mortality rates of cerebrovascular disease, infectious respiratory disease, and suicide are relatively high ^(17)^. Although previous studies have reported an association between specific disease morbidity rates and HLE ^(18)^, the association between the corresponding mortality rates and HLE is not well documented. HLE combines information on mortality and morbidity; thus, verifying its association with cause-specific mortality rates is crucial.

As mortality rates vary greatly with age, it is difficult to compare them among various regions with different age structures. The standardized mortality ratio (SMR) is used to describe whether a specific population is more, less, or equally likely to die when compared with a standard/reference population ^(19), (20), (21)^. The SMR uses an indirect adjustment method to compare or evaluate mortality due to several causes of death within a given area against a common standard.

For effective health promotion measures, the local characteristics of each administrative district must be identified and considered during planning. The Japanese medical system has been developed according to the specific needs of the population in different regions, and secondary medical care facilities are an important aspect of the organizational structure. In Japan, secondary medical care is provided in 344 districts. Secondary medical care facilities are responsible for general inpatient care and cater to the health needs of several municipalities. As such, the medical care system has been developed according to the specific needs of the population in these areas. On the basis of the Medical Service Act, these areas, through their respective prefectural governments, are required to provide general medical supplies and adequate medical resources (e.g., hospital beds and clinic facilities) ^(22), (23)^. However, significant population size differences at a regional level may reduce statistical accuracy when examining the data at a local level. These differences may inadvertently affect the prioritization of regional health services and resource allocation for specific health needs. This study focused on secondary medical care areas to examine the factors related to HLE at a regional level to ensure maximum accuracy in documenting the effect of the causes of mortality on HLE. Thus, this study aimed to determine the association between HLE and the SMRs of major diseases in secondary healthcare service areas in Japan.

## Materials and Methods

### Data and data sources

To calculate HLE, most health promotion plans at the national and prefectural levels in Japan use daily life limitations; however, many health promotion and data health plans at the municipal level, which provide more specific health measures for residents, use long-term care insurance data to calculate HLE. Therefore, in this study, HLE was calculated using long-term care insurance data. In addition, some municipalities have a small population size, which may reduce statistical accuracy when examining the data at a municipal level ^(24)^. Therefore, in this study, analyses were performed based on secondary medical care areas to examine factors related to HLE on a regional basis to ensure maximum accuracy. We evaluated HLE as of 2017 based on the latest data available in the Japanese resident registration database ^(25)^. The administrative processing data for all residents, excluding their personal information, were used. Mortality data were obtained from the total number of deaths reported in the 2016-2018 vital statistics ^(26)^. In this study, “healthy” was defined as a period in which daily activities were not restricted and “unhealthy” was defined as a period in which daily activities were restricted. Data on the degree of long-term care required were obtained from the “long-term care insurance service report.” The Japanese long-term care system is divided into levels 1 to 5 based on the individual long-term care needs, as certified by the Japanese long-term care insurance system ^(27), (28)^. Data on unhealthy individuals, including long-term care levels 2 (almost bedridden) to 5, were obtained from the 2017 long-term care insurance data ^(29)^. In this study, those at level 1 or lower or level 2 or higher were categorized as “requiring almost no long-term care” (healthy) and “needing long-term care” (unhealthy), respectively.

### Outcome: HLE

There are various methods to calculate HLE ^(24), (30), (31)^. The Sullivan method, adopted by Healthy Japan 21, is used to calculate HLE using data on mortality rates and the percentage of the healthy population ^(14)^. The percentage of the healthy population is calculated using the duration of independence in daily life activities.

We estimated HLE stratified by sex using the Sullivan method, which considers age-specific mortality and life with disabilities. In this method, by applying the age-specific prevalence of a particular unhealthy state to a life table function (i.e., the number of years of life in each age interval), the total life expectancy is calculated as the number of years of life spent in good health (i.e., HLE).

### Predictor: SMRs

When mortality rates vary widely by age and cannot be compared directly between regions with different age structures, SMR is preferred for comparison. SMR, an indirect age-adjusted method, is advantageous because the variance is relatively small; the calculation is easy and can be performed even when the number of deaths or the population size is small ^(10), (11)^.

SMR compares the actual number of deaths observed with the expected number of deaths and is calculated by applying the reference mortality rate (number of deaths per 100,000 population) to the target area ^(21)^. If SMR is ≥100, the mortality rate is considered higher than the Japanese average; if it is less than 100, the mortality rate is considered lower than the Japanese average. SMR is often used for regional comparisons because it can be easily calculated using the reference mortality rate and the population of the target region. The data on SMRs in this study were analyzed using demographic statistics for 5 years from 2013 to 2017 ^(32)^. We used SMRs for the leading causes of death in Japan in our analysis ^(32)^. All data on SMRs for major causes of death, including malignant neoplasm, heart disease, cerebrovascular disease, pneumonia, liver disease, renal failure, unexpected accidents, and suicide, were obtained from the database of the Ministry of Health, Labor and Welfare of Japan. The International Classification of Diseases codes were used to classify data on mortality.

### Ethical approval

All data used in this study are national public data that are openly accessible. Hence, this study was exempted from the requirement for ethics committee approval and informed consent.

### Statistical analyses

HLE and SMR values were calculated at the level of secondary medical areas. First, the mean and standard deviation values of the calculated variables were obtained, and values exceeding the extreme limits (>three standard deviations) were excluded from the analysis as outliers. To ensure uniformity, if more than one variable exceeded three standard deviations, that variable was excluded from all analyses. Thus, for men, 12 of 344 regions were excluded, leaving 332 regions for analysis. For women, 8 of 344 regions were excluded, leaving 336 regions for analysis. To analyze the association of various mortality rates with HLE and unhealthy life expectancy, multiple regression analysis was performed separately for men and women, with SMRs for major diseases (malignant neoplasm, heart disease, cerebrovascular disease, pneumonia, liver disease, renal failure, unexpected accidents, and suicide) as the explanatory variable and HLE as the objective variable. Two models were used for prediction. In “Model 1,” each predictor was entered individually to assess its univariate association with each outcome. In “Model 2,” all predictors were entered simultaneously. Multicollinearity was then evaluated using the variance inflation factor (VIF). The VIF value was <2, indicating no multicollinearity among the predictors. The Statistical Package for Social Sciences version 27 (IBM Corp., Armonk, NY, USA) was used for all statistical analyses.

## Results

### Descriptive statistics for HLE and SMR

The average healthy periods were 79.24 (0.85) and 83.76 (0.62) years for men and women, respectively (Table 1). The descriptive statistics for the SMRs for major diseases are described in Table 2.

**Table 1.** Healthy and Unhealthy Life Expectancies.

	*Men*				*Women*			
	*M*	*SD*	*Min*	*Max*	*M*	*SD*	*Min*	*Max*
Healthy life expectancy (years)	79.24	0.85	76.90	81.36	83.76	0.62	81.99	85.45
Unhealthy life expectancy (years)	1.40	0.17	0.90	1.90	3.11	0.33	2.19	4.39

Abbreviations: M, mean; SD, standard deviation; Max, maximum value; Min, minimum value

**Table 2.** Descriptive Statistics for Standardized Mortality Ratios.

	*Men*				*Women*			
	*M*	*SD*	*Min*	*Max*	*M*	*SD*	*Min*	*Max*
Malignant neoplasm	100.24	7.53	82.28	127.71	99.22	6.45	81.49	122.10
Heart disease	101.34	14.25	59.69	140.77	102.01	13.29	67.98	157.28
Cerebrovascular disease	104.61	16.87	69.79	159.07	104.39	17.39	70.91	163.32
Pneumonia	101.07	16.45	54.96	158.16	100.80	21.28	47.60	178.40
Liver disease	96.12	15.86	59.28	164.60	98.60	16.43	59.45	165.14
Renal failure	103.85	18.55	66.59	168.11	103.28	19.56	59.91	198.50
Unexpected accidents	109.96	21.44	62.42	181.92	105.23	17.73	59.34	156.60
Suicide	107.98	17.06	73.85	168.53	101.95	16.39	62.15	185.86

Abbreviations: M, mean; SD, standard deviation; Max, maximum value; Min, minimum value

### Association between SMR and HLE

In Model 1 for men (Table 3), all SMRs for major diseases (malignant neoplasm, heart disease, cerebrovascular disease, pneumonia, liver disease, renal failure, unexpected accidents, and suicide) were negatively associated with HLE. In Model 2, the SMRs for malignant neoplasm (β = −0.342, *p* < 0.001), heart disease (β = −0.215, *p* < 0.001), cerebrovascular disease (β = −0.281, *p* < 0.001), pneumonia (β = −0.090, *p* = 0.008), liver disease (β = −0.202, *p* < 0.001), and suicide (β = −0.146, *p* < 0.001) were negatively associated with HLE. In Model 2, unhealthy life expectancy tended to be longer when the mortality rates for heart and liver diseases were high. In addition, the higher the mortality rates for cerebrovascular disease and pneumonia were, the shorter was the unhealthy life expectancy.

**Table 3.** Association between Standardized Mortality Ratios and Healthy Life Expectancy in Men.

	*Model 1^a^*					*Model 2^b^*				
	*B*	*SE*	*β*	*p*	*Adjusted R^2^*	*B*	*SE*	*β*	*p*	*Adjusted R^2^*
*Healthy life expectancy*										
Malignant neoplasm	−0.072	0.005	−0.635	<0.001	0.402	−0.039	0.004	−0.342	<0.001	
Heart disease	−0.029	0.003	−0.489	<0.001	0.237	−0.013	0.002	−0.215	<0.001	
Cerebrovascular disease	−0.029	0.002	−0.580	<0.001	0.334	−0.014	0.002	−0.281	<0.001	
Pneumonia	−0.024	0.003	−0.463	<0.001	0.212	−0.005	0.002	−0.090	0.008	0.738
Liver disease	−0.022	0.003	−0.420	<0.001	0.174	−0.011	0.002	−0.202	<0.001	
Renal failure	−0.022	0.002	−0.471	<0.001	0.220	−0.002	0.002	−0.045	0.209	
Unexpected accidents	−0.014	0.002	−0.352	<0.001	0.121	−0.003	0.001	−0.067	0.065	
Suicide	−0.028	0.002	−0.562	<0.001	0.314	−0.007	0.002	−0.146	<0.001	
*Unhealthy life expectancy*										
Malignant neoplasm	−0.004	0.001	−0.158	0.004	0.022	−0.001	0.002	−0.030	0.657	
Heart disease	0.001	0.001	0.064	0.245	0.001	0.002	0.001	0.199	0.001	
Cerebrovascular disease	−0.002	0.001	−0.175	0.001	0.028	−0.002	0.001	−0.187	0.004	
Pneumonia	−0.002	0.001	−0.219	<0.001	0.045	−0.002	0.001	−0.213	0.001	0.115
Liver disease	0.001	0.001	0.054	0.326	0.000	0.001	0.001	0.118	0.035	
Renal failure	−0.001	0.000	−0.128	0.020	0.013	−0.001	0.001	−0.084	0.204	
Unexpected accident	−0.001	0.000	−0.074	0.180	0.002	0.000	0.001	0.039	0.556	
Suicide	−0.001	0.001	−0.128	0.019	0.013	0.000	0.001	−0.034	0.639	

Abbreviations: B, unstandardized coefficient; SE, standard error; β, standardized coefficient; *p*, *p*-value Note: We evaluated healthy life expectancy and unhealthy life expectancy as of 2017 based on the latest data available in the Japanese resident registration database. The data excluding personal information for the administrative processing of all residents were used. Mortality data were obtained from the total number of deaths reported in the 2016-2018 vital statistics. Furthermore, this was a cross-sectional analysis. ^a^Model 1: Each predictor was entered individually to assess its univariate association with each outcome. ^b^Model 2: All predictors were entered simultaneously.

In Model 1 for women (Table 4), the SMRs for malignant neoplasm, heart disease, cerebrovascular disease, pneumonia, liver disease, renal failure, and suicide, excluding that for unexpected accidents, were negatively associated with HLE. In Model 2, the SMRs for malignant neoplasm (β = −0.422, *p* < 0.001), heart disease (β = −0.277, *p* < 0.001), cerebrovascular disease (β = −0.186, *p* < 0.001), and suicide (β = −0.112, *p* = 0.013) were negatively associated with HLE. In addition, in Model 2, the higher the mortality rates for cerebrovascular disease and pneumonia were, the shorter was the unhealthy life expectancy.

**Table 4.** Association between Standardized Mortality Ratios and Healthy Life Expectancy in Women.

	*Model 1^a^*					*Model 2^b^*				
	*B*	*SE*	*β*	*p*	*Adjusted R^2^*	*B*	*SE*	*β*	*p*	*Adjusted R^2^*
*Healthy life expectancy*								
Malignant neoplasm	−0.045	0.005	−0.471	<0.001	0.219	−0.041	0.004	−0.422	<0.001	
Heart disease	−0.020	0.002	−0.424	<0.001	0.177	−0.013	0.002	−0.277	<0.001	
Cerebrovascular disease	−0.008	0.002	−0.229	<0.001	0.049	−0.007	0.002	−0.186	<0.001	
Pneumonia	−0.009	0.002	−0.312	<0.001	0.094	−0.001	0.001	−0.046	0.354	0.425
Liver disease	−0.011	0.002	−0.283	<0.001	0.077	−0.003	0.002	−0.089	0.069	
Renal failure	−0.008	0.002	−0.242	<0.001	0.056	−0.001	0.002	−0.023	0.638	
Unexpected accidents	0.003	0.002	0.086	0.115	0.004	0.001	0.002	0.041	0.353	
Suicide	−0.008	0.002	−0.213	<0.001	0.042	−0.004	0.002	−0.112	0.013	
*Unhealthy life expectancy*								
Malignant neoplasm	0.000	0.003	−0.004	0.935	−0.003	0.003	0.003	0.052	0.368	
Heart disease	0.000	0.001	−0.013	0.818	−0.003	0.003	0.002	0.111	0.077	
Cerebrovascular disease	−0.004	0.001	−0.216	<0.001	0.044	−0.005	0.001	−0.269	<0.001	
Pneumonia	−0.002	0.001	−0.146	0.007	0.018	−0.003	0.001	−0.181	0.004	0.085
Liver disease	−0.001	0.001	−0.057	0.301	0.000	−0.001	0.001	−0.051	0.409	
Renal failure	−0.002	0.001	−0.090	0.098	0.005	−0.001	0.001	−0.055	0.372	
Unexpected accident	−0.002	0.001	−0.127	0.020	0.013	−0.002	0.001	−0.086	0.126	
Suicide	0.001	0.001	0.041	0.459	−0.001	0.002	0.001	0.096	0.092	

Abbreviations: B, unstandardized coefficient; SE, standard error; β, standardized coefficient; *p*, *p*-value Note: We evaluated healthy life expectancy and unhealthy life expectancy as of 2017 based on the latest data available in the Japanese resident registration database. The data excluding personal information for the administrative processing of all residents were used. Mortality data were obtained from the total number of deaths reported in the 2016-2018 vital statistics. Furthermore, this was a cross-sectional analysis. ^a^Model 1: Each predictor was entered individually to assess its univariate association with each outcome. ^b^Model 2: All predictors were entered simultaneously.

## Discussion

The association between mortality rates of specific diseases and HLE remains unclear. Therefore, this study aimed to investigate the association between HLE and SMRs for major diseases that cause death. When all predictors were entered simultaneously into the model, the associations between HLE and malignant neoplasm, heart disease, cerebrovascular disease, and suicide were significant in men and women. In particular, the SMRs of malignant neoplasms were strongly associated with HLE. The adjusted coefficients of determination were also calculated for both sexes, with a higher value obtained for men than for women (men, adjusted R^2^ = 0.738; women, adjusted R^2^ = 0.425). Therefore, focusing on these diseases may contribute to HLE extension and may reduce health disparity. Our findings specifically suggest that the health plans of local governments should prioritize cancer screening and smoking cessation as preventive measures for cancer deaths, especially among men ^(33), (34), (35), (36), (37), (38)^.

Our results are consistent with reports of previous studies documenting associations between major diseases and life expectancy in other countries ^(39), (40), (41), (42), (43)^. Malignant neoplasm, cardiovascular disease, cerebrovascular disease, pneumonia, and liver disease are major chronic diseases in Japan, which may affect the quality of life and may lengthen the period of ill health ^(44), (45)^. In addition, suicide was significantly associated with HLE in our results. The suicide rate in Japan is higher in men than in women ^(46)^. Mental and physical health problems (e.g., depression and physical illness) are often listed as causes and motivations for suicide, which may influence the duration of poor health. In 2020, 21,081 cases of suicide were reported in Japan (912 cases more than the count reported in the previous year); this was the first increase observed in 11 years, making suicide prevention an urgent need ^(41)^. In particular, in Model 1 for men (Table 3), the adjusted R^2^ values for malignant neoplasm (0.402) and cardiovascular disease (0.334), which have high mortality rates, were high. Interestingly, the adjusted R^2^ for suicide (0.314) was equally high as those for malignant neoplasm and cardiovascular disease, although the mortality rate for suicide was lower than that for other causes of death. Suicide is often a result of deteriorating mental health, which is also associated with several factors that affect HLE ^(47), (48), (49), (50)^, and has a profound effect on HLE and not just life expectancy.

Regarding unhealthy life expectancy, it is expected that the adjusted R^2^ for unhealthy life expectancy is lower than that for HLE. However, in relative terms, when considering the adjusted R^2^ of Model 1 of unhealthy life expectancy, the adjusted R^2^ for pneumonia in men (0.045) and that for cerebrovascular disease in women (0.044) were higher than the adjusted R^2^ for other causes of death and were negatively associated with unhealthy life expectancy. Pneumonia and cerebrovascular disease are highly associated with mortality, predominantly cerebrovascular disease. The burden of stroke is disproportionately high in women as they live longer than men and are more likely to experience cerebrovascular disease than other diseases. Therefore, cerebrovascular disease in women may have a greater impact than other diseases ^(51), (52), (53), (54)^. Consequently, pneumonia in men and cerebrovascular disease in women may shorten unhealthy life expectancy and HLE (i.e., shortening life expectancy itself).

The causes of death in Japan by disease are ranked in decreasing order as follows: malignant neoplasm at 28%, followed by heart disease at 15% and cerebrovascular disease at 8%, accounting for approximately half of the total number of deaths ^(16)^. Japanese people have the longest life expectancy and HLE among both sexes, particularly among women. Notably, Japan has the lowest mortality rates associated with cancer and ischemic heart disease worldwide ^(21), (22)^. Although reducing mortality rates is difficult, empirical findings on lifestyle factors and regional characteristics related to mortality risk and efforts to deal with these factors are accumulating ^(55), (56), (57)^. On the basis of the study results, we expect that the focus of local governments on health promotion plans for these particular diseases may improve HLE estimates. Specifically, because the SMRs of malignant neoplasms were strongly associated with HLE, improving cancer screening rates and reducing smoking rates can contribute to lowering cancer incidence and mortality rates ^(33), (34), (35), (36), (37), (38)^. Thus, such a medical policy may contribute to HLE extension and health disparity reduction. In addition, this study was validated at the regional level for each secondary healthcare area. In Japan, public health centers have contributed greatly to improving local resident health; they are accessible in almost all secondary medical care areas. Public health centers are involved in various healthcare policies, such as cancer control and suicide prevention, and potentially play a major role in extending HLE.

This study has some limitations. First, secondary data collected from various sources were analyzed. Although the data collection systems are robust, the limitations of using secondary data apply to this study. Second, this study model was designed to accommodate a specific geographic location (Japan), allowing room for selection bias. The study results are partially consistent with those of previous studies conducted in other countries. However, it is difficult to generalize the results to other countries as the data analyzed were exclusive to the Japanese population. Third, the mortality indicator used, underlying cause of death, is the primary cause of death. However, because almost everyone in Japan dies at an old or very old age, it is likely that most people have several comorbidities at a given time and some may even get ill and are dependent on others. Therefore, it is difficult to identify the primary cause of death as being related to HLE. Fourth, the lack of correlation between the cause-specific SMR and unhealthy life expectancy for each cause of death analyzed (and several causes of death in the analysis of HLE) is a limitation to the usefulness of this study from a public health perspective. The issue of co-/multimorbidity, which is not discussed extensively in this manuscript, may explain why no (strong) association was found, as well as a low level of direct causal association between poor health (in terms of care needs) and outcome in the case of suicide and unexpected accidents. We calculated HLE using long-term care insurance data. However, this may not be the best indicator for mental health-related causes of death. Finally, regarding unexpected accidents and suicide, the population density of Tokyo is approximately 90 times that of Hokkaido, and there are differences of approximately 1.5 times between the shortest and longest amounts of daylight. Therefore, it might not be fair to compare the SMRs ^(26), (32)^. Our results were not adjusted for population density and daylight time.

In conclusion, we reported the association between the SMRs of major diseases and HLE at the secondary medical area level in Japan. The SMRs among men and women for malignant neoplasm, heart disease, cardiovascular disease, and suicide were negatively associated with HLE. In particular, the SMRs of malignant neoplasms were significantly and negatively associated with HLE. Therefore, focusing on these diseases may contribute to HLE extension and health disparity reduction. Extending HLE may also affect the total life expectancy.

## Article Information

### Conflicts of Interest

None

### Sources of Funding

This work was supported by the Health Labor Sciences Research Grants (grant numbers 19FA1012, 19FA2001, and 22FA0901).

### Acknowledgement

We are grateful to the Health Labor Sciences Research Council for providing the grant to conduct this study.

### Author Contributions

NK was responsible for the acquisition of funds. RH and TO were responsible for the research design and investigation, methodology, resources, software used, validation and visualization of data, and writing the original draft of the manuscript. TO was responsible for supervising the study. NK was responsible for project administration. TM, JA, KK, and NK were responsible for manuscript reviewing and editing. All authors have approved the final article.

### Approval by Institutional Review Board (IRB)

All data used in this study are national public data that are openly accessible. Hence, this study was exempted from ethics committee approval and informed consent requirements.
